# Neuroanatomical Localization of the Vestibular Cortex: A Case Report

**DOI:** 10.7759/cureus.41061

**Published:** 2023-06-27

**Authors:** Sana Gulraiz, Muhammad Fawad Ishfaq, Taha F Rasul, Adnan Qureshi

**Affiliations:** 1 School of Public Health, West Virginia University School of Medicine, Morgantown, USA; 2 Neurology, University of Missouri Hospital, Columbia, USA; 3 Infectious Diseases, University of Miami Miller School of Medicine, Miami, USA

**Keywords:** stroke, limbic lobe, vertigo, dizziness, vestibular cortex

## Abstract

Vertigo, a symptom of illusory movement, is caused by asymmetry of the vestibular system. The vestibular system consists of the vestibular labyrinth, cranial nerve VIII, brainstem vestibular nuclei, cerebellum, ocular motor nuclei, spinal cord, and less well-defined cerebral projections. In this day and age of artificial intelligence, machine learning, advanced imaging, and cutting-edge research in the field of neurology, the exact cortical control of vestibular function is still uncharted. A 45-year-old woman with a past medical history of labyrinthitis about 4.5 years ago (resolved) presented to hospital due to severe dizziness, emesis, and mild vertical diplopia for the past few days. Her symptom of dizziness i.e. room spinning was continuous without any postural component. MRI of the brain revealed a small stroke in the left hippocampal area, more specifically alveus of hippocampus. The patient was started on dual antiplatelet therapy and atorvastatin for secondary stroke prevention. Follow-up visit as an outpatient at one-month post hospital discharge was unremarkable without any recurrence of vertigo symptoms. We believe this may indicate that the limbic lobe has a much larger role in vestibular functioning than previously thought, and may control more vestibular operations than any other central nervous system area.

## Introduction

The vestibular system is responsible for balance, both stationary and in motion. This is important because of the constant need for postural/oculomotor stability and spatial awareness. The vestibular function has been thought to be controlled by various areas in the frontal, temporal and parietal lobes. It is important to characterize the control center of vestibular function so that the etiology of balance pathologies is further clarified. Recognition of the exact cortical control would also be fruitful in yielding the site of lesion via a clinical examination, particularly in stroke patients. 

Epidemiologic studies have shown that central neurological causes are responsible for up to 11% of patient complaints related to dizziness and up to 4% of complaints related to vertigo [1]. A patient’s history, neurological examination, and imaging studies typically provide the most important metrics in differentiating between the peripheral and central causes of vertigo. The most common central causes of vertigo are typically cerebrovascular in nature [2]. Other pathologies such as migraines, posterior fossa tumors, and neuropsychiatric disorders can manifest as central vertigo [2].

## Case presentation

The patient, a 45-year-old female with a history of hypothyroidism, essential hypertension, obesity, and resolved labyrinthitis requiring intra-auditory steroids in the left ear about 4.5 years ago, presented to the emergency department with dizziness, emesis, and double vision. She stated that for the past few days she had been having symptoms of dizziness where she felt that the room was spinning. This was evident by the near-continuous nature of the vertigo episode(s) upon admission. She tried to use a scopolamine patch with only mild relief of symptoms. Her past history of labyrinthitis had also led to left-ear tinnitus and mild hearing loss. She also had a posterior left-sided headache extending down the left side of the neck, but did not have migrainous characteristics (no sensitivity to any sensory input such as sound/light/smell, unilateral, pulsating, worse with physical activity). She did not report taking alcohol or narcotics, having a cold, stiff neck, ear fullness, ear itching, ear pain, or head trauma before the vertigo attack. 

The patient initially saw her primary care physician for the symptoms and underwent a rapid magnetic resonance imaging (MRI) brain which showed punctate restricted diffusion within the left hippocampus. Her primary care physician recommended her to go to the emergency department immediately upon receiving the results. Further evaluation of the patient in the emergency department revealed that she was hypertensive with blood pressure of 176/107 mmHg; other vital signs were within normal limits. Physical examination was significant for an imbalance of gait in all directions and mild vertical diplopia which was persistent in all directions without any change with gaze. Cerebellar signs were all intact on examination. The National Institute of Health Stroke Scale (NIHSS) was calculated to be at zero because it does not cover the above-mentioned positive findings as described in this patient. 

Further workup while inpatient included: computed tomography (CT) head had no evidence of acute intracranial abnormality; CT angiography head and neck showed no evidence of significant intracranial abnormality, with patent carotid and vertebro-basilar systems, transthoracic echocardiography showed ejection fraction of 60%, no wall motion abnormalities, valvular abnormalities or right-to-left shunt. The patient was started on dual antiplatelet therapy and atorvastatin for secondary stroke prevention. Complete resolution was seen seven days after symptom onset. The follow-up outpatient visit at a stroke clinic was one-month post hospital discharge where patient reported no further episodes of dizziness.

## Discussion

**Figure 1:**
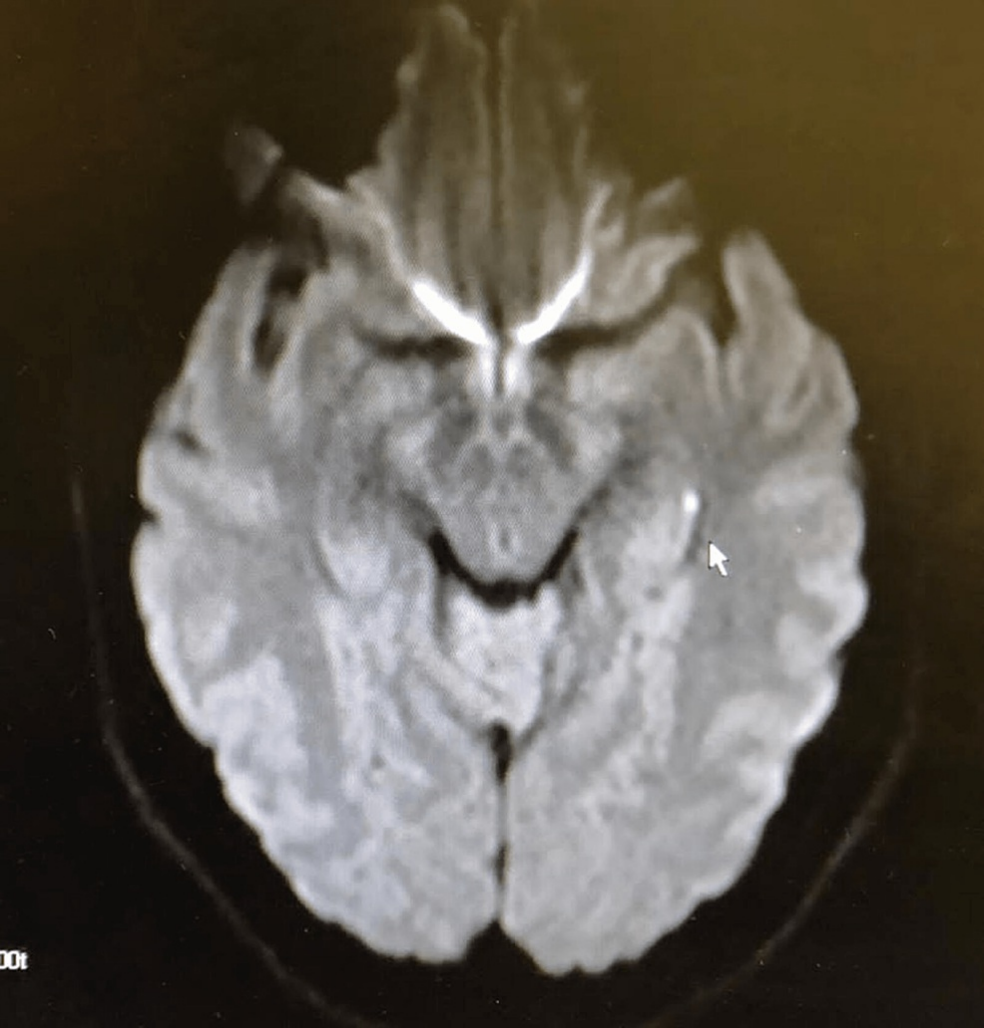
T2 star weighted-diffusion-weighted image (DWI) cross-section at the level of the pons, showing a small, white pinpoint alvear-hippocampal stroke (directly next to the mouse cursor), located near the insular region. This localized lesion in our patient, who experienced severe vertigo, gives a clue to the location of the vestibular system control.

Evident from this case was the occurrence of vestibular symptoms with a small left hippocampal lesion in the area covered by the alveus. Vestibular symptoms have been reported in various studies with excitation of certain cortical regions [3]. The limbic lobe is the arc-shaped medial cortical region that connects the frontal, parietal, occipital, and temporal lobes bilaterally [4]. As such, it includes most of the cingulate gyrus and passes in close proximity to various regions of the hippocampus such as fimbriae. The limbic lobe and has traditionally been thought to primarily mediate olfaction and lesions in the lobe display predominantly cortical symptoms [5]. Our patient represents a rare situation where the alvear pathway of the hippocampus underwent a very small stroke (Figure 1), which ended up causing pronounced symptoms of vertigo. Such a finding heavily implies vestibular control within the alvear hippocampal area. Therefore, in terms of localizing the vestibular cortex, a very specific lesion such as the one seen in this case would produce symptoms specific to the region of control. Even though the vestibular cortex has been thought to occur in a right-hemispheric dominant pattern, our patient’s left-sided lesion also means that there may be bilateral control of the vestibular cortex instead of being localized to one hemisphere [6]. 

Vertigo is a very common complaint in the general population, usually referred to as “dizziness” or “sensation of room spinning”. It results from asymmetric involvement of the vestibular system. Vertigo is of two main types - central and peripheral. Central vertigo occurs when there is a problem within the vestibular nuclei in the brainstem, or their projections or further connections to the brain, be it the cerebrum or cerebellum [7]. Peripheral vertigo results from a more upstream lesion in the sensory pathway. Cerebrovascular disease causes 3% to 6% of vertigo in general. Physical therapy or vestibular therapy is key in re-engaging areas of the nervous system in a stroke patient. This is most critical in the early stages after stroke, due to the positive effects of exercise on the release of hippocampal brain-derived neurotrophic factor (BDNF) which demonstrated memory function recovery [8]. It remains to be seen, however, how BDNF expression post-stroke would vary in hippocampal strokes such as the one seen in this case. 

The parieto-insular vestibular cortex was first described to contain areas of vestibular perception in Java monkeys in 1990 [9]. Over the years, the vestibular system has been postulated to have many control centers in different parts of the brain and extensive integration into various cortices has been found in various primates. 

Imaging and stimulatory methods have been used to investigate the location of the vestibular cortex in humans, as mentioned in recent literature reviews. Two meta-analyses, from 2012 discuss that the opercular-insular complex, the inferior parietal cortex, the cingulate cortex and the pre-motor cortex are important areas of vestibular processing [10,11]. There is also an important role of the cingulate sulcus visual area. 

Other, more recent studies also provide direction. Vestibular symptoms can be present in acute hemispheric strokes, with a predilection towards the right cortical hemisphere. The temporo-perisylvian vestibular cortex, which was a lateral cortical temporoparietal area, was postulated by Kahane et al. to correspond with monkey parietoinsular vestibular cortex as the site where cortical stimulation could easily give rise to vestibular symptoms. Of note, the majority of vestibular sites in the cortex (19 out of 41) were found to be in the temporal lobe, followed by parietal (14), frontal (5), occipital (2), and insular (1) [12]. Parietoinsular vestibular cortex+ was referred to in a recent study, as a combination of at least two areas (including parietoinsular vestibular cortex and PIC, which is the posterior insular cortex) that were responsible in humans for the core of vestibular processing [13]. This means that multiple central nervous system (CNS) locations play important roles in vestibular circuitry, even though the exact central control area is not well-understood. 

Another observation is that stroke patients with acute hemispheric lesions do not mostly present with vertigo and a possible explanation has been compensation by other areas of the cortex and thus suppression of symptoms [14,15]. Ten cases of nonepileptic vertigo after middle cerebral artery stroke have been reported, and affected areas have been found consistently in the parietoinsular vestibular cortex/posterior retroinsular cortex and parietal vestibular cortex [16]. But one specific region is yet to be identified as the core region for vestibular processing. It seems likely that the alvear hippocampal area of the limbic lobe plays a much larger role than previously thought in the regulation of the vestibular system, and may even be the central processing area for the vestibular cortex. Intentional lesions in the alveus such as disconnecting surgeries for the management of epilepsy have been found to improve the symptoms of attention deficit hyperactivity disorder (ADHD), suggesting a potential role in attention and cognition, in addition to involvement in vestibular control [17]. Due to the sheer volume of neural circuitry passing through the hippocampal area, it may also be possible that our patient’s lesion specifically affected vestibular neurons and left other systems intact. 

Better understanding of the areas of vestibular control can allow for more accurate clinical suspicion. This will ideally lead to precise interventions and therapies to efficiently diagnose and treat strokes. If a stroke patient presents with dizziness and balance issues, a hippocampal alvear infarct can be included on the differential as a potential cause. 

## Conclusions

The exact region of cortical control for vestibular function is still not well understood, with studies showing a wide variety of cortical areas involved. It is important to recognize where certain balance issues originate from, especially in patients with stroke. Clinically identifying an area of stroke could be done if one observes complaints related to balance in a patient. Severe vertigo can happen in a patient with a small alvear-hippocampal ischemic stroke. The limbic lobe has a more vital role than previously thought, particularly in the function of the vestibular cortex, and should be considered in patients with new-onset vertigo in the context of a stroke.
